# Supplementary material for: A pre-registered short-term forecasting study of COVID-19 in Germany and Poland during the second wave
Source: Nat Commun. 2021 Aug 27;12:5173. doi: 10.1038/s41467-021-25207-0 (PMC8397791; doi:10.1038/s41467-021-25207-0)
Supplement: Supplementary file 1 — Supplementary Information [file 41467_2021_25207_MOESM1_ESM.pdf]

Supplementary Materials for Bracher et al. (2021): A pre-registered short-term forecasting study of COVID-19 in Germany and Poland during the second wave

Supplementary Note 1 Additional time series plots

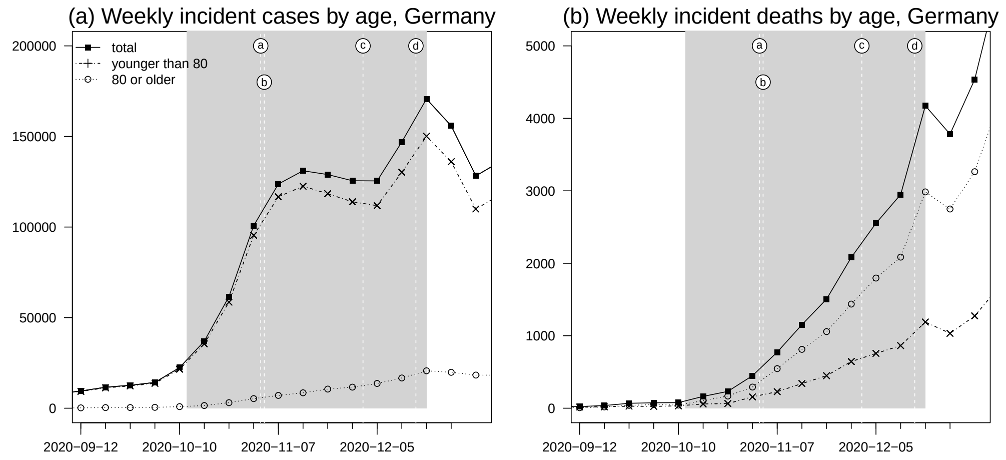

Supplementary Figure 1: **a** Weekly incident COVID-19 cases and **b** deaths in Germany, pooled and stratified by age below and above 80 years. The time period covered by our study is highlighted in grey. Events marked by letters a – d are explained in Figure 1.

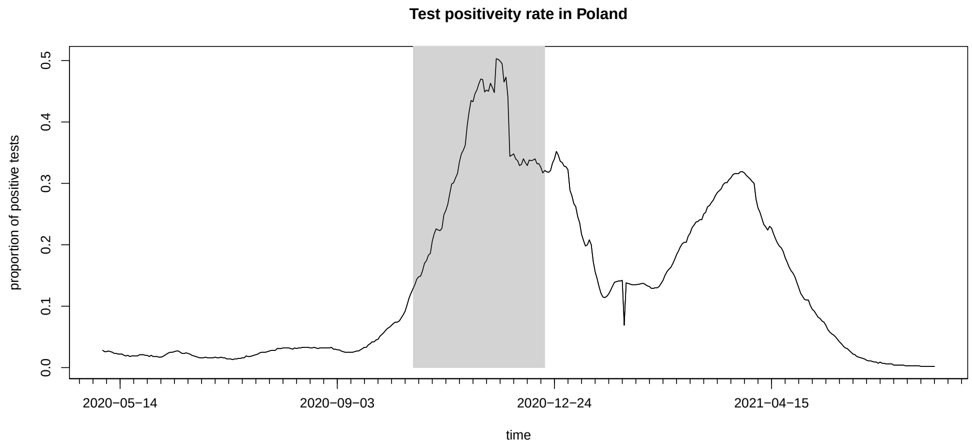

Supplementary Figure 2: Smoothed test positivity rates in Poland. The time period covered by our study is highlighted in grey.

Test positivity data were downloaded from https://ourworldindata.org/coronavirus-testing and originate from

Hasell, J., Mathieu, E., Beltekian, D. et al. A cross-country database of COVID-19 testing. *Sci Data* 7, 345 (2020)

Supplementary Note 2 Detailed description of baseline forecasts

We here describe the three baseline forecasts mentioned in the main manuscript in more detail.

`KIT-baseline` Denote the quantity of interest on the incidence scale by X_t. The corresponding quantity on the cumulative scale is denoted by $Y_t = \sum_{s \leq t} X_t$. The one-week-ahead forecast for X_{t+1} is given by a negative binomial distribution with mean X_t and overdispersion parameter ψ. Due to the skewness of the negative binomial distribution this implies that the predictive median is slightly smaller than X_t. The overdispersion parameter is estimated from the last five available observations using a maximum likelihood approach, i.e. by maximizing

$$\sum_{i=0}^{4} \log \pi(X_{t-i} \mid X_{t-i-1}, \psi) \tag{3}$$

with respect to ψ, where $\pi(\cdot \mid X_{t-i-1}, \psi)$ is the probability mass function of a negative binomial distribution with mean X_{t-i-1} and overdispersion parameter ψ. For technical reasons we replace any mean of a negative binomial distribution which would equal zero by 0.2. The two- to four-week-ahead forecasts are simply set to the same distribution as the one-week-ahead forecast.

To obtain forecasts on the cumulative scale we assume independence between $X_{t+1}, X_{t+2}, X_{t+3}$ and X_{t+4}. As the sum of independent random variables following negative binomial distributions with the same overdispersion parameter follows again a negative binomial distribution, $Y_{t+1}, Y_{t+2}, Y_{t+3}$ and Y_{t+4} follow shifted negative binomial distributions with overdispersion parameter $\psi, 2\psi, 3\psi$ and 4ψ, respectively.

`KIT-extrapolation_baseline` We assume again a (conditional) negative binomial distribution, but with mean $\lambda_{t+1} = \alpha X_t$ rather than just X_t. The parameter α is estimated from the last three observed values in the following way:

- If the last three observations are ordered, i.e. $X_{t-2} < X_{t-1} < X_t$ or $X_{t-2} > X_{t-1} > X_t$ we let

$$\alpha = \frac{X_t}{X_{t-1}}, \tag{4}$$

  which corresponds to simple multiplicative extrapolation.

- Otherwise we let $\alpha = 1$, so that the predictive mean λ_{t+1} equals the last observation X_t.

The idea behind this distinction is that the model should only use trends if they have manifested for at least two weeks. The overdispersion parameter is estimated by maximizing

$$\sum_{i=1}^{5} \log \pi(X_{t-i} \mid \lambda_{t-i}, \psi), \tag{5}$$

with respect to ψ (keeping the value α entering into $\lambda_{t-i} = \alpha X_{t-i-1}$ constant at the value chosen as described above). Note that we do not use the last observation X_t here as by construction (if the last three observations are ordered) $X_t = \lambda_t$.

We then sample 100,000 paths $(X_{t+1}, X_{t+2}, X_{t+3}, X_{t+4})$ from this model and obtain forecast quantiles for both incident and cumulative quantities from these samples.

`KIT-time_series_baseline` We fit an exponential smoothing model with multiplicative errors and without seasonality to the last 12 observations on the incidence scale. The `R` (26) command is

```
forecast::ets(ts, model="MMN")
```

using the `forecast` package (40) (version 8.12). As noted in the main text, this specification is taken from (41). As in the previous section we proceed by sampling paths from this model and computing predictive quantiles from them.

Supplementary Note 3 Descriptions of submitted forecasts

We provide more extensive descriptions of the models listed in Table 3, including details on inference approaches and computation times.

`CovidAnalytics-DELPHI` Predictions for future cases are obtained from a heavily modified SEIR model. New states are added to account for cases that went undetected, while quarantined and hospitalized patients are separated. The infection rate is corrected with a nonlinear curve that represents the cumulative effect of governmental and societal response (which is assumed to change according to the magnitude of the outbreak). Key parameters for the disease are fixed using a meta-analysis conducted by the CovidAnalytics group of over 150 parameters while epidemiological parameters are fitted to historical death counts and detected cases. The epidemiological parameters are fitted using a moving time window with truncated Newton and simulated annealing to effectively capture the latest changes in the epidemic trends. Uncertainty intervals are generated using re-sampling of the out-of-sample error for the predictions two weeks ago by fitting the historical out-of-sample error to a normal distribution. Then we assume that the incremental estimates for each week have random errors of such magnitude that are independent (and additive) by week. Generating the forecasts for one country takes a few minutes on a standard laptop.

`epiforecasts-EpiExperts` The `EpiExpert` model represents predictions from experts and non-experts that were submitted through an R Shiny application (app.crowdforecastr.org). Participants could select a distribution family and specify the median and spread of the predictive distribution by dragging points or adjusting numeric input values. Available distributions were several transformations of a normal distribution (normal, log-normal, 3rd-, 5th, and 7th-power normal). From every predictive distribution, 23 quantiles were obtained. Lastly, an ensemble was formed by aggregating predictions using a quantile-wise mean. Participation increased over time from around 3 to around 5–10 weekly forecasters. To inform their forecasts, participants could use any data they liked. The data shown in the app was data from the ECDC, RKI and the Polish Ministry of Health. Participants were shown additional information from ourworldindata.org.

`epiforecasts-EpiNow2` `EpiNow2` is an exponential growth model that uses a time-varying R_t trajectory to forecast latent infections, and then convolves these infections using known delays to observations, via a negative binomial model coupled with a day of the week effect. It makes limited assumptions and is not tuned to the specifics of COVID-19 in Germany and Poland beyond epidemiological details such as literature estimates of the generation time, incubation period and the population of each country. The reproductive number R_t is assumed to remain static after the respective last observed values.

Each forecast target was fit independently for each model using Markov-chain Monte Carlo (MCMC) using the Stan software and RStan interface (https://mc-stan.org/rstan/). A minimum of 4 chains were used with a warmup of 250 samples and 2000 samples total post warmup. Forecast intervals were calculated from the generated MCMC samples, aggregating daily posterior samples to the required weekly scale. Forecast generation for all targets, both national and subnational, took not more than 2 hours using 16 CPU cores.

`FIAS FZJ-Epi1Ger` This is an extended version of the well-established SEIR (susceptible – exposed – infectious – removed) model, i.e. a deterministic approach based on a system of ordinary differential equations. Mixing in the population is assumed to be homogeneous, but infectiousness varies across three possible compartments, namely, asymptomatic undetected, symptomatic undetected and detected cases. Detection can occur both in an early stage, that is, during latent phase (E, assumed not yet infectious) or when the patient is already infectious (asymptomatic/symptomatic). Undetected infections lead to undetected recoveries, whereas detected cases may either recover or die. We do not explicitly incorporate a reporting delay in recording fatalities. Daily new detected cases and reported deaths are used for model calibration. Transmission, detection and death rates are assumed to be piecewise constant in time and fitted using reported data (time series data from RKI/ECDC). The fitting algorithm is based on Monte Carlo sampling and minimization of sum of squared residuals, evaluating results with the Akaike information criterion. Parameter values obtained from the fit of the most recent interval are used for model predictions (incident/cumulative cases and deaths). The model is not age-stratified and does not take into account advance knowledge on

mitigation measures to be applied, e.g. to reduce contact rates in the population. To quantify the uncertainty of forecasts, the model fits obtained via Monte Carlo sampling of the parameter space are collected in histograms according to their Akaike weights. Predictive quantiles are calculated from these weighted histograms. The calculations take about 10 to 15 minutes on a single core of a workstation, but can be accelerated considerably by partly re-using results of recent model runs.

`ICM-agentModel` The model aims to represent the social structure of the Polish population at the level of the individual citizens and their social contacts. It is spatially structured and contains representations of different contexts. The model follows the development of the epidemic on a geographical grid with a resolution of 1km and through physical contact in various contexts of social life. It currently contains representations of households, workplaces, kindergardens, schools, universities, travel, streets and public places. The approach can serve for short-term prediction of the development of the epidemic and for the exploration of various scenarios. These can shed light on the effects of newly introduced policies and non-pharmaceutical interventions. The model features a detailed demographic stratification where each agent has an attribute of age and gender. The likelihood of severe symptoms, hospitalization, ICU treatment and death depends on age. Agents of different age appear in different contexts (schools, workplaces, travel, etc.) with different probabilities. The model is fitted via a Bayesian inference approach. Data inputs include publicly available epidemiological data (number of cases, deaths etc) from the Polish Ministry of Health as well as more detailed data available via the Polish National Institute for Public Health (time of hospitalization, age, symptoms, contact tracing etc). Computations are performed on a Cray-XC40 supercomputer. The generation of forecast for Poland takes 1 hour of computing time on 10 nodes (node: Intel Xeon E5-2690 v3 2.6 GHz 2 x 12 cores, hyperthreading x2, 128 GB RAM).

`imperial-ensemble2` To generate forecasts at a horizon of seven days, an unweighted ensemble of three models is used:

- Model 1 assumes a conditional Poisson distribution and the renewal equation

$$I_t \sim \text{Pois}\left(R_t \sum_{s=0}^{t} I_{t-s} w_s\right), \tag{6}$$

  where I_t are deaths reported on day t. Here, the serial interval distribution w is a gamma distributon with mean 6.48 days and a standard deviation of 3.83 days. The instantaneous reproduction number R_t is estimated via a maximum likelihood approach from the last 10 days (jointly with the incidence prior to the 10 day window). Forecasts are obtained by simulating stochastic realisations of the renewal equation from the end of the calibration period.

- Model 2 optimises the choice of time window over which the reproduction number is assumed to be constant for estimating. The optimal window is one which minimises the accumulated predictive error (APE) in 1-step ahead predictions over the entire time series. Estimates of reproduction numbers using the optimal time window are then used to project forward using the renewal equation.

- Model 3 uses both the reported number of cases and deaths. The reported cases are weighted with a reporting to death delay distribution to obtain the largest potential number of deaths, if all reported cases were to die. The observed number of deaths is used to estimate an observed fatality ratio. Forward projections are then obtained by sampling from a binomial distribution with the weighted case count and estimated fatality ratio.

For each model, we generate 10000 samples from the posterior distribution of reproduction number and obtain 10000 simulations of forward projections. Uncertainty intervals are provided by the quantiles of the posterior distribution. Computations require approximately 10 minutes on an 8 GB Macbook Pro.

`ITWW-county_repro` Reproduction numbers on the county level are estimated via a small area approach where reproduction numbers are modeled as random variables. First we sample for every county reproduction numbers for the past week and compute their mean. This value is held constant over one simulation and

we sample from the reproduction equation to generate daily future cases on the county level. These are aggregated to the state and country level. Finally, for Germany, we account for the delay with which these cases appear in the ECDC dataset as our reproduction numbers are based on the official RKI dataset. To forecast deaths we sample on the state level the age of every case and use case-fatality ratios for every age group to forecast which cases will later be marked as death; the delay until death is sampled from a reporting-to-death distribution. We then aggregate the deaths from the state level to the country level. The model contains a detailed age stratification. For Germany we use age-stratified attack-rates and case-fatality ratios which we estimate from the official RKI data. The age groups we consider are: 0–4, 5–14, 15–34, 35–59, 60–79, 80+ and unknown. For Poland we consider age groups from 0 to 100 by year (101 age groups). Generation of forecasts takes less than 10 minutes on a standard laptop.

`LANL-GrowthRate` The model makes predictions about the future, unconditional on particular intervention strategies. It consists of two processes. The first process is a statistical model of how the number of COVID-19 infections changes over time. The second process maps the number of infections to the reported data. We model the growth of new cases as the product of a dynamic growth parameter and the underlying numbers of susceptible and infected cases in the population at the previous time step, scaled by the size of the state's starting susceptible population. The growth parameter can be thought of as the transmissibility of the virus in that state on that date and is a weighted regression between the trend in the growth rate over the past 42 days and a growth rate that would keep the number of new daily confirmed cases constant. The weights of these two components are dynamically tuned to the observed data. To model new deaths in the population, we assume that a fraction of the 1, 2, 3, 4, or 5-week moving average of the daily confirmed cases will die. The model learns both the moving average window and the case fatality fraction that best fits the historical observations.

`LeipzigIMISE-SECIR` An adapted mechanistic epidemiological model of the SECIR (susceptible – exposed – carriers – infected – recovered) type is integrated into Input-Output Non-Linear Dynamical Systems (IO-NLDS) serving as hidden layers, i.e. the true dynamics cannot be observed directly. The model contains an asymptomatic compartment, a compartment of patients requiring intensive care, and most of the compartments are divided into three sub-compartments to represent time delays. Changing factors of the system due to non-pharmaceutical interventions, changing age-structure of the infected population, and changes in testing policies are imposed as inputs to the system. Parameters are then estimated by a knowledge synthesis process considering parameter ranges derived from external studies and public data. Specifically, a Bayesian inference approach is taken to estimate time-varying parameters. Public data is translated to model outputs not identical but related to hidden states of the model. The model is fitted to data by a full information approach. The Stochastic Approximation Expectation Maximization (SAEM) algorithm is used to estimate model parameters by minimizing the negative log-likelihood of the observations. The number of updates of time-dependent variables is determined via the Bayesian information criterion (BIC). After determination of residual errors of parameters via SAEM, MCMC chains are simulated in order to sample possible alternative parameter sets around the optimal solution from the distribution, determined by the constrained negative log-likelihood function. Generation of forecasts requires several hours on a standard desktop computer.

`MIMUW-StochSEIR` The model uses an extension of the SEIR (susceptible – exposed – infected – recovered) approach. The key developments to account for the specifics of COVID-19 are: (i) inclusion of the daily number of tests into the model; (ii) addition of a state representing undiagnosed infected and undiagnosed recovered patients; (iii) a Bayesian inference approach. The main parameters of this extended model are assigned prior distributions with hyperparameters based on the literature or preliminary analyses using deterministic SEIR models. The posterior distributions are computed using the Monte Carlo Metropolis–Hastings algorithm. The final parameter estimates are then given by the posterior means, and the uncertainty intervals by respective quantiles. The typical computation (3000 steps with 1000 steps of the burn-in phase) takes roughly 4 hours on a 2.3 GHz Dual-core Intel Core i5.

`MOCOS-agent1` This is an agent based model based on heterogeneous random network structures for potentially infectious contacts. The network structure is defined by sets of context and feature-dependent

non-symmetric kernels. The dynamics is time-continuous, event driven microsimulation. It takes into account census data on household composition, age distribution, work places etc. The model includes contact tracing, both classic and app-based, testing and quarantine. All relevant duration times like incubation time, time to hospitalization and time to testing are sampled from distributions based on empirical data. Spatial structures are implemented but not used at the moment for country wide forecasts. We take into account changes in delay distribution over time, e.g in time from symptom onset to reporting. Even more importantly, changes in testing strategies and their impact on the dark figure of cases are taken into account

Inference is done using a mixture of Baysian inference, maximum likelihood methods and Monte Carlo search. Uncertainty intervals are generated via a likelihood distribution over an ensemble of suitable sample paths involving different parameters. For each relevant parameter configuration, 100 sample paths are generated and weighted.

Computations are done in parallel on the Lower Silesia (Poland) high performance super cluster and depending on size of parameter space to be searched can take more than 24 hours

`UCLA-SuEIR` The SuEIR model is a variation of the SEIR (susceptible – exposed – infected – recovered) model that features the following compartments (with the total size of the population denoted by N):

- S_t: The number of susceptible individuals at time t, i.e. individuals who can still acquire the disease.
- E_t: The number of individuals who have already been infected but have not been tested/diagnosed with the disease. Unlike in the classic SEIR model this group can also cause new infections.
- I_t: The number of individuals who are infected and have received a positive test.
- R_t: The number of individuals who have been infected and received a positive test and have since either recovered or died.
- u_t: The unobserved number of individuals who have been infected, but not received a positive test, and have since recovered or died.

These compartments are linked via the following system of differential equations:

$$\frac{\mathrm{d}S_t}{\mathrm{d}t} = -\frac{\beta(I_t + E_t)S_t}{N}, \quad \frac{\mathrm{d}E_t}{\mathrm{d}t} = \frac{\beta(I_t + E_t)S_t}{N} - \sigma E_t, \quad \frac{\mathrm{d}I_t}{\mathrm{d}t} = \mu\sigma E_t - \gamma I_t, \tag{7}$$

$$\frac{\mathrm{d}R_t}{\mathrm{d}t} = \gamma I_t, \quad \frac{\mathrm{d}u_t}{\mathrm{d}t} = (1-\mu)\sigma E_t, \tag{8}$$

where β is the contact rate between the susceptible and infectious (i.e. I_t and E_t) groups, γ is the rate at which individuals leave the detected case compartment (due to recovery or death), μ is the discovery rate and σ is the ratio of cases in the exposed compartments that are either confirmed as infectious or dead/recovered without confirmation. To generate predictions of fatalities, a time-varying ratio r_t of deaths and removals due to recovery is estimated. The model is fit to reported cases and fatalities via a logarithmic-type mean squared error approach and gradient based optimization.

`USC-SIkJalpha` Forecasts are generated using an epidemic model called `SIkJalpha`, a preliminary version of which has been successfully used during the DARPA Grand Challenge 2014. The model can consider the effect of many complexities of the epidemic process and yet be simplified to a few parameters that are learned using fast linear regressions. Therefore, the approach can learn and generate forecasts extremely quickly. The model is able to quickly adapt to changing trends, and the variations in parameters during different times/policies enable the generation of different scenarios such as what would happen if we were to disregard social distancing suggestions. For each state, hospitalizations are modelled as a separate compartment, as a linear function of recent cases with heterogeneous rates. This means that for a hyper-parameter J, those who were infected at time $t-1$ to $t-J$ will have a separate rate from those infected at $t-(J+1)$ to $t-2J$, and so on. Death forecasts are generated in a similar fashion. For long-term forecasts (more than a few days in the future), cases are predicted first based on the `SIkJalpha` model, which forms the input to hospitalization prediction. While changing trends are accounted for by putting more emphasis on recently

seen data, it is assumed that the trends will remain the same in the future for point forecasts. The approach attempts to account for changing trends in the future in the quantile forecasts by modeling the empirical errors using a random forest.

`SDSC-ISG_TrendModel` Forecasts are based on the reported numbers of cases and deaths at the country or regional level. No information about measures or changes in policies is used for forecasting. The modelling substantially relies on trend estimation of the time series of the number of daily cases/deaths. To account for non-stationary weekly seasonality, outliers, missing data and delayed reports, and reliably estimate the underlying trend for each of the time series, we use a robust seasonal trend decomposition model based on LOESS (LOcally Estimated Scatterplot Smoothing). In order to better adapt to the changes in the data, trends are estimated locally in overlapping time series subintervals, while the global smooth trend estimate is a pointwise weighted combination of the overlapping local models. To predict daily cases and deaths we use linear extrapolation of the estimated smooth trend either on the original or on the logarithmic scale.

Supplementary Note 4 Details on truth data sources

The different truth data are publicly available in the following locations:

- Daily data compiled by ECDC until 14 December are available at https://www.ecdc.europa.eu/en/publications-data/download-todays-data-geographic-distribution-covid-19-cases-worldwide
- Daily data compiled by the Center for Systems Science and Engineering at Johns Hopkins University (25, JHU) are available at https://github.com/CSSEGISandData/COVID-19/tree/master/csse_covid_19_data
- Daily data from Robert Koch Institute were extracted from https://npgeo-corona-npgeo-de.hub.arcgis.com/datasets/dd4580c810204019a7b8eb3e0b329dd6_0. Note that the data provided there are on a different time scale than used in this article (a mix of symptom onset and date of reporting to local authorities). Data by reporting date to the national authorities were generated by comparing data sets made available on subsequent days. The generated data set (available in the Forecast Hub platform) is in agreement with the ECDC data up to 14 December.
- Daily data from the Polish Ministry of Health were extracted from a widely used public spread sheet maintained by Michal Rogalski:
  https://docs.google.com/spreadsheets/u/2/d/1ierEhD6gcq51HAm433knjnVwey4ZE5DCnu1bW7PRG3E.
  These data are in agreement with the ECDC data up to 14 December.

All truth data time series, including historical versions, are also available in the folder https://github.com/KITmetricslab/covid19-forecast-hub-de/tree/master/data-truth of our repository.

Supplementary Note 5 Sources on changes in non-pharmaceutical interventions and testing regimes

We here provide sources for the dates of interventions shown in Figure 1.

Poland: Government interventions are largely documented on the respective governmental web site and the Twitter channel of the Polish Ministry of Health (in Polish):

- https://www.gov.pl/web/koronawirus/100-dni-solidarnosci-w-walce-z-covid-19
- https://twitter.com/MZ_GOV_PL.

Specific news items on mentioned interventions/events:

- Four symptoms required for test: Ministerstwo Zdrowia przekazało zasady zlecania testów na koronawirusa, Wprost, 23 September 2020, https://www.wprost.pl/koronawirus-w-polsce/10368723/ministerstwo-zdrowia-przekazalo-zasady-zlecania-testow-na-koronawirusa.html (last accessed 22 December 2020).
- Only one out of four symptoms required for test: Dlaczego lekarz odmawia skierowania na test na COVID-19? Medonet, 5 November 2020, https://www.medonet.pl/koronawirus/koronawirus-w-polsce,kiedy-lekarz-moze-odmowic-skierowania-na-test-na-koronawirusa,artykul,26303647.html (last accessed 22 December 2020)
- Bulk reporting of 22,000 cases on 24 November: Rozbiezności w statystykach koronawirusa. 22 tys. przypadków będą doliczone do ogólnej liczby wyników, Forsal, 23 November 2020, https://forsal.pl/lifestyle/zdrowie/artykuly/8017628,rozbieznosci-w-statystykach-koronawirusa-22-tys-przypadkow-beda-doliczone-do-ogolnej-liczby-wynikow.html (last accessed 22 December 2020)
- High test positivity and suspected under-ascertainment: Polish doctors fear high rate of positive COVID tests show pandemic worse than it appears, J. Plucinska, Reuters, 1 December 2020, https://www.reuters.com/article/us-health-coronavirus-poland-cases/polish-doctors-fear-high-rate-of-positive-covid-tests-show-pandemic-worse-than-it-appears-idUSKBN28B54Q (last accessed 22 December 2020)

Germany: A chronicle of the most important events (in German) can be found on the web site of the German Ministry of Health and in a report issued by Robert Koch Institute:

- https://www.bundesgesundheitsministerium.de/coronavirus/chronik-coronavirus.html
- Schilling, J., Buda, S., Fischer, M., Goerlitz, L., Grote, U., Haas, W., Hamouda, O., Prahm, K., and Tolksdorf, K. (2021). Retrospektive Phaseneinteilung der COVID-19-Pandemie in Deutschland bis Februar 2021. *Epidemiologisches Bulletin*, 2021/15:3–12. Available at http://dx.doi.org/10.25646/8149.

Specific news items on mentioned interventions/events:

- Semi-lockdown from 2 November onwards: Coronavirus: Germany to impose one-month partial lockdown, Deutsche Welle, 28 October 2020, https://www.dw.com/en/coronavirus-germany-to-impose-one-month-partial-lockdown/a-55421241 (last accessed 22 December 2020)
- New testing strategy announced: SARS-CoV-2-Diagnostik: RKI passt Testempfehlungen an, Ärzteblatt, 3 November 2020, https://www.aerzteblatt.de/nachrichten/118001/SARS-CoV-2-Diagnostik-RKI-passt-Testempfehlungen-an (last accessed 22 December 2020)
- Reinforced rules from 1 December onwards: Was gilt wo im Corona-Dezember? Tagesschau, 1 December 2020, https://www.tagesschau.de/inland/corona-plan-bundeslaender-beschluss-103.html (last accessed 22 December 2020)
- Full lockdown starting on 16 December: Lockdown in Deutschland – Das sind die Corona-Regeln. Tagesschau, 13 December 2020, https://www.tagesschau.de/inland/corona-regeln-lockdown-101.html (last accessed 22 December 2020)

Supplementary Note 6 Availability and delays of forecasts

Supplementary Table 1: Availability of forecasts by model, target and forecast horizon.

Each entry describes up to which forecast horizon (in weeks) forecasts for incident cases, cumulative cases, incident deaths and cumulative deaths were made available (numbers in this order and separated by semicolons). Asterisks indicate that forecasts were only available on Wednesday or later rather than before Tuesday 3pm.

Germany

	2020-10-12	2020-10-19	2020-10-26	2020-11-02	2020-11-09	2020-11-16	2020-11-23	2020-11-30	2020-12-07	2020-12-14
epiforecasts-EpiExpert	4; 4; 4; 4	4; 4; 4; 4	4; 4; 4; 4	4; 4; 4; 4	4; 4; 4; 4	4; 4; 4; 4	4; 4; 4; 4	4; 4; 4; 4	4; 4; 4; 4	4; 4; 4; 4
epiforecasts-EpiNow2	4; 4; 4; 4*	4; 4; 4; 4	4; 4; 4; 4	4; 4; 4; 4	4; 4; 4; 4	4; 4; 4; 4	4; 4; 4; 4	4; 4; 4; 4	4; 4; 4; 4	4; 4; 4; 4
FIAS_FZJ-Epi1Ger	4; 4; 4; 4	4; 4; 4; 4	4; 4; 4; 4	4; 4; 4; 4	4; 4; 4; 4	4; 4; 4; 4	4; 4; 4; 4	4; 4; 4; 4	4; 4; 4; 4	4; 4; 4; 4
Imperial-ensemble2	-; -; 1; 1*	-; -; 1; 1*	-; -; 1; 1*	-; -; 1; 1*	-; -; 1; 1*	-; -; 1; 1*	-; -; 1; 1*	-; -; 1; 1*	-; -; 1; 1*	-; -; 1; 1
ITWW-county_repro	4; 4; 4; 4	4; 4; 4; 4	4; 4; 4; 4	4; 4; 4; 4	4; 4; 4; 4	4; 4; 4; 4	4; 4; 4; 4	4; 4; 4; 4*	4; 4; 4; 4	4; 4; 4; 4
KIT-baseline	4; 4; 4; 4	4; 4; 4; 4	4; 4; 4; 4	4; 4; 4; 4	4; 4; 4; 4	4; 4; 4; 4	4; 4; 4; 4	4; 4; 4; 4	4; 4; 4; 4	4; 4; 4; 4
KIT-extrapolation_baseline	4; 4; 4; 4*	4; 4; 4; 4*	4; 4; 4; 4*	4; 4; 4; 4*	4; 4; 4; 4*	4; 4; 4; 4*	4; 4; 4; 4*	4; 4; 4; 4*	4; 4; 4; 4*	4; 4; 4; 4
KIT-time_series_baseline	4; 4; 4; 4*	4; 4; 4; 4*	4; 4; 4; 4	4; 4; 4; 4	4; 4; 4; 4	4; 4; 4; 4	4; 4; 4; 4	4; 4; 4; 4	4; 4; 4; 4	4; 4; 4; 4
inverse_wis_ensemble	4; 4; 4; 4*	4; 4; 4; 4*	4; 4; 4; 4*	4; 4; 4; 4*	4; 4; 4; 4*	4; 4; 4; 4	4; 4; 4; 4	4; 4; 4; 4	4; 4; 4; 4	4; 4; 4; 4
mean_ensemble	4; 4; 4; 4*	4; 4; 4; 4*	4; 4; 4; 4	4; 4; 4; 4	4; 4; 4; 4*	4; 4; 4; 4	4; 4; 4; 4	4; 4; 4; 4	4; 4; 4; 4	4; 4; 4; 4
median_ensemble	4; 4; 4; 4*	4; 4; 4; 4*	4; 4; 4; 4	4; 4; 4; 4	4; 4; 4; 4*	4; 4; 4; 4	4; 4; 4; 4	4; 4; 4; 4	4; 4; 4; 4	4; 4; 4; 4
LANL-GrowthRate	-; 4; -; 4	-; 4; -; 4	-; 4; -; 4	4; 4; 4; 4*	4; 4; 4; 4	4; 4; 4; 4	4; 4; 4; 4	4; 4; 4; 4	4; 4; 4; 4	4; 4; 4; 4
LeipzigIMISE-SECIR	4; 4; 4; 4	4; 4; 4; 4	4; 4; 4; 4	4; 4; 4; 4*	4; 4; 4; 4	4; 4; 4; 4*	4; 4; 4; 4	4; 4; 4; 4	4; 4; 4; 4*	4; 4; 4; 4*
MIT_CovidAnalytics-DELPHI	4; -; 4; 4	4; -; 4; 4	4; -; 4; 4	4; -; 4; 4	4; -; 4; 4	4; -; 4; 4	4; -; 4; 4	-; -; -; -	-; -; -; -	4; -; 4; 4
SDSC_ISG-TrendModel	1; 1; 1; 1	1; 1; 1; 1	1; 1; 1; 1	1; 1; 1; 1	1; 1; 1; 1	1; 1; 1; 1	1; 1; 1; 1	1; 1; 1; 1	1; 1; 1; 1	1; 1; 1; 1*
UCLA-SuEIR	4; 4; 4; 4*	4; 4; 4; 4*	4; 4; 4; 4	4; 4; 4; 4	4; 4; 4; 4	4; 4; 4; 4	4; 4; 4; 4	4; 4; 4; 4	4; 4; 4; 4	4; 4; 4; 4*
USC-SIkJalpha	4; 4; 4; 4	4; 4; 4; 4	4; 4; 4; 4	4; 4; 4; 4	4; 4; 4; 4	4; 4; 4; 4	4; 4; 4; 4	4; 4; 4; 4	4; 4; 4; 4	4; 4; 4; 4

Poland

	2020-10-12	2020-10-19	2020-10-26	2020-11-02	2020-11-09	2020-11-16	2020-11-23	2020-11-30	2020-12-07	2020-12-14
epiforecasts-EpiExpert	4; 4; 4; 4	4; 4; 4; 4	4; 4; 4; 4	4; 4; 4; 4	4; 4; 4; 4	4; 4; 4; 4	4; 4; 4; 4	4; 4; 4; 4	4; 4; 4; 4	4; 4; 4; 4
epiforecasts-EpiNow2	4; 4; 4; 4*	4; 4; 4; 4	4; 4; 4; 4	4; 4; 4; 4	4; 4; 4; 4	4; 4; 4; 4	4; 4; 4; 4	4; 4; 4; 4	4; 4; 4; 4	4; 4; 4; 4
ICM-agentModel	-; -; 4; 4	-; -; -; -	-; -; 4; 4*	-; -; -; -	-; -; 4; 4	-; -; 4; 4	4; 4; 4; 4	4; 4; 4; 4	4; 4; 4; 4*	4; 4; 4; 4
Imperial-ensemble2	-; -; 1; 1*	-; -; 1; 1*	-; -; 1; 1*	-; -; 1; 1*	-; -; 1; 1*	-; -; 1; 1*	-; -; 1; 1*	-; -; 1; 1*	-; -; 1; 1*	-; -; 1; 1
ITWW-county_repro	4; 4; 4; 4	4; 4; 4; 4	4; 4; 4; 4	4; 4; 4; 4	4; 4; 4; 4	4; 4; 4; 4	4; 4; 4; 4	4; 4; 4; 4*	4; 4; 4; 4	4; 4; 4; 4
KIT-baseline	4; 4; 4; 4	4; 4; 4; 4	4; 4; 4; 4	4; 4; 4; 4	4; 4; 4; 4	4; 4; 4; 4	4; 4; 4; 4	4; 4; 4; 4	4; 4; 4; 4	4; 4; 4; 4
KIT-extrapolation_baseline	4; 4; 4; 4*	4; 4; 4; 4*	4; 4; 4; 4*	4; 4; 4; 4*	4; 4; 4; 4*	4; 4; 4; 4*	4; 4; 4; 4*	4; 4; 4; 4*	4; 4; 4; 4*	4; 4; 4; 4
KIT-time_series_baseline	4; 4; 4; 4*	4; 4; 4; 4*	4; 4; 4; 4	4; 4; 4; 4	4; 4; 4; 4	4; 4; 4; 4	4; 4; 4; 4	4; 4; 4; 4	4; 4; 4; 4	4; 4; 4; 4
inverse_wis_ensemble	4; 4; 4; 4*	4; 4; 4; 4*	4; 4; 4; 4*	4; 4; 4; 4*	4; 4; 4; 4*	4; 4; 4; 4	4; 4; 4; 4	4; 4; 4; 4	4; 4; 4; 4	4; 4; 4; 4
mean_ensemble	4; 4; 4; 4*	4; 4; 4; 4*	4; 4; 4; 4	4; 4; 4; 4	4; 4; 4; 4*	4; 4; 4; 4	4; 4; 4; 4	4; 4; 4; 4	4; 4; 4; 4	4; 4; 4; 4
median_ensemble	4; 4; 4; 4*	4; 4; 4; 4*	4; 4; 4; 4	4; 4; 4; 4	4; 4; 4; 4*	4; 4; 4; 4	4; 4; 4; 4	4; 4; 4; 4	4; 4; 4; 4	4; 4; 4; 4
LANL-GrowthRate	-; 4; -; 4	-; 4; -; 4	-; 4; -; 4	4; 4; 4; 4*	4; 4; 4; 4	4; 4; 4; 4	4; 4; 4; 4	4; 4; 4; 4	4; 4; 4; 4	4; 4; 4; 4
MIMUW-StochSEIR	-; -; -; -	-; -; -; -	-; -; -; -	-; -; -; -	-; -; -; -	4; 4; 4; 4*	4; 4; 4; 4*	4; 4; 4; 4*	4; 4; 4; 4*	4; 4; 4; 4
MIT_CovidAnalytics-DELPHI	4; -; 4; 4	-; -; -; -	4; -; 4; 4	4; -; 4; 4	4; -; 4; 4	4; -; 4; 4	4; -; 4; 4	4; -; 4; 4	4; -; 4; 4	4; -; 4; 4
MOCOS-agent1	4; 4; 4; 4	4; 4; 4; 4*	4; 4; 4; 4	4; 4; 4; 4	4; 4; 4; 4*	4; 4; 4; 4	4; 4; 4; 4	4; 4; 4; 4	4; 4; 4; 4	4; 4; 4; 4
SDSC_ISG-TrendModel	1; 1; 1; 1	1; 1; 1; 1	1; 1; 1; 1	1; 1; 1; 1	1; 1; 1; 1	1; 1; 1; 1	1; 1; 1; 1	1; 1; 1; 1	1; 1; 1; 1	1; 1; 1; 1*
USC-SIkJalpha	4; 4; 4; 4	4; 4; 4; 4	4; 4; 4; 4	4; 4; 4; 4	4; 4; 4; 4	4; 4; 4; 4	4; 4; 4; 4	4; 4; 4; 4	4; 4; 4; 4	4; 4; 4; 4

Most forecasts from Imperial-ensemble2 were only made available retrospectively to the Forecast Hub, but had previously been shown in real time on the web dashboard of the Imperial team.

Supplementary Note 7 Additional results for one- and two-week-ahead forecasts

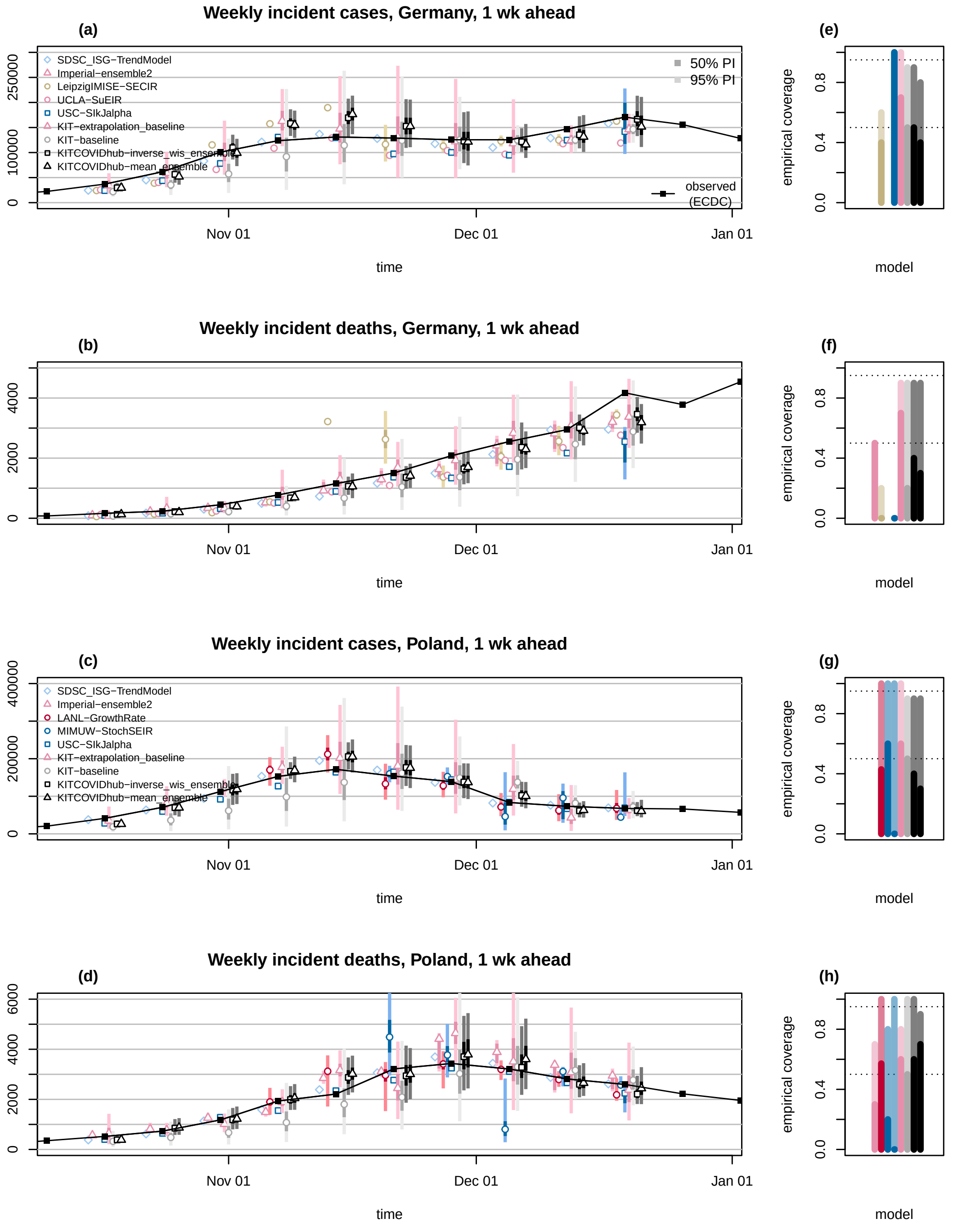

Supplementary Figure 3: **Additional one-week-ahead forecasts.** One-week-ahead forecasts of incident cases and deaths in Germany (**a, b**) and Poland (**c, d**). Displayed are predictive medians, 50% and 95% prediction intervals (PIs) for models not shown in Figure 2. Coverage plots (**e–h**) show the empirical coverage of 95% (light) and 50% (dark) prediction intervals.

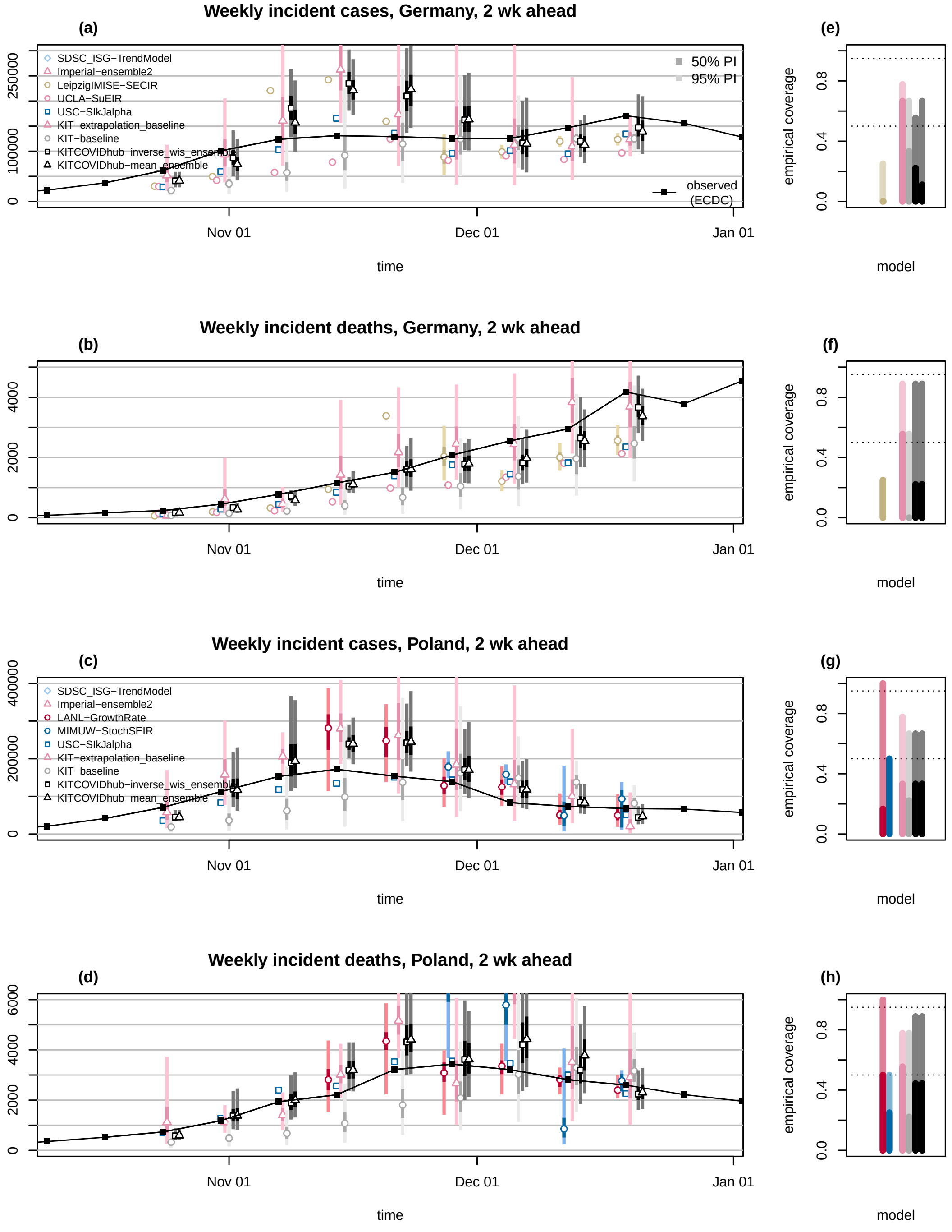

Supplementary Figure 4: **Additional two-week-ahead forecasts.** Two-week-ahead forecasts of incident cases and deaths in Germany (**a, b**) and Poland (**c, d**). Displayed are predictive medians, 50% and 95% prediction intervals (PIs) for models not shown in Figure 3. Coverage plots (**e–h**) show the empirical coverage of 95% (light) and 50% (dark) prediction intervals.

Supplementary Table 2: Detailed summary of forecast evaluation for Germany (based on JHU data)

Germany, cases																
	1 wk ahead inc				2 wk ahead inc				1 wk ahead cum				2 wk ahead cum			
Model	AE	WIS	$C_{0.5}$	$C_{0.95}$	AE	WIS	$C_{0.5}$	$C_{0.95}$	AE	WIS	$C_{0.5}$	$C_{0.95}$	AE	WIS	$C_{0.5}$	$C_{0.95}$
epiforecasts-EpiExpert	13,351	9,470	3/10	7/10	33,588	24,996	2/9	3/9	13,351	9,470	3/10	7/10	46,506	34,234	2/9	4/9
epiforecasts-EpiNow2	9,879	6,413	6/10	8/10	35,103	26,003	4/9	6/9	9,879	6,413	6/10	8/10	43,106	31,285	5/9	7/9
FIAS_FZJ-Epi1Ger	7,377	4,720	5/10	10/10	29,886	20,438	4/9	7/9	14,775	9,326	3/10	7/10	38,614	27,427	4/9	6/9
ITWW-county_repro	34,825	29,077	0/10	2/10	63,812	52,478	0/9	2/9	34,476	28,729	0/10	2/10	100,873	83,619	0/9	1/9
LANL-GrowthRate	38,679*	22,973*	4/7	7/7	79,788*	43,165*	2/6	6/6	39,734	27,405	4/10	7/10	120,334	81,370	2/9	5/9
LeipzigIMISE-SECIR	20,064		1/5	3/5	51,576		0/4	1/4	36,089	31,838	0/10	1/10	93,828	84,209	1/9	2/9
MIT_CovidAnalytics-DELPHI	40,959*	29,499*	2/8	4/8	82,336*	63,549*	0/7	2/7								
SDSC_ISG-TrendModel	14,173								14,173							
UCLA-SuEIR	28,331				50,970				28,331				76,523			
USC-SIkJalpha	21,935		1/1	1/1	34,150				23,474		1/1	1/1	53,480			
KIT-baseline	21,980	15,006	4/10	8/10	35,913	28,029	3/9	5/9	21,980	15,006	4/10	8/10	53,840	42,428	3/9	5/9
KIT-extrapolation_baseline	12,851	10,487	7/10	10/10	37,194	26,323	5/9	7/9	12,851	10,487	7/10	10/10	47,861	34,846	7/9	7/9
KIT-time_series_baseline	14,910	10,950	6/10	9/10	43,955	28,311	5/9	8/9	14,910	10,950	6/10	9/10	59,721	38,684	4/9	8/9
KITCOVIDhub-inverse_wis_ensemble	14,205	8,992	3/10	9/10	42,759	27,897	1/9	6/9	13,525	8,813	4/10	8/10	53,689	34,975	2/9	7/9
KITCOVIDhub-mean_ensemble	17,484	10,712	3/10	8/10	42,910	27,776	2/9	6/9	16,171	10,397	3/10	7/10	57,842	37,415	1/9	6/9
KITCOVIDhub-median_ensemble	12,271	8,001	4/10	9/10	38,316	25,784	3/9	7/9	13,277	8,962	4/10	7/10	50,155	34,504	2/9	6/9

Germany, deaths																
	1 wk ahead inc				2 wk ahead inc				1 wk ahead cum				2 wk ahead cum			
Model	AE	WIS	$C_{0.5}$	$C_{0.95}$	AE	WIS	$C_{0.5}$	$C_{0.95}$	AE	WIS	$C_{0.5}$	$C_{0.95}$	AE	WIS	$C_{0.5}$	$C_{0.95}$
epiforecasts-EpiExpert	236	156	4/10	7/10	390	268	3/9	6/9	236	156	4/10	7/10	545	350	2/9	7/9
epiforecasts-EpiNow2	159	108	5/10	8/10	357	218	4/9	7/9	159	108	5/10	8/10	485	296	5/9	7/9
FIAS_FZJ-Epi1Ger	307	268	2/10	4/10	579	484	1/9	3/9	244	206	0/10	3/10	779	655	2/9	4/9
Imperial-ensemble2	306	225	2/10	5/10					303	222	2/10	5/10				
ITWW-county_repro	420	403	0/10	2/10	594	536	0/9	1/9	419	402	0/10	2/10	924	852	0/9	2/9
LANL-GrowthRate	234*	148*	4/7	7/7	500*	353*	2/6	5/6	218	149	4/10	7/10	658	493	3/9	5/9
LeipzigIMISE-SECIR	653		0/5	1/5	815		1/4	1/4	1,199	1,024	0/10	1/10	2,247	1,867	0/9	1/9
MIT_CovidAnalytics-DELPHI	499*	378*	2/8	3/8	401*	313*	1/7	5/7	473*	353*	2/8	3/8	635*	533*	1/7	3/7
SDSC_ISG-TrendModel	409								409							
UCLA-SuEIR	508				884				508				1,281			
USC-SIkJalpha	540		0/1	0/1	657				552		0/1	0/1	1,066			
KIT-baseline	531	291	0/10	9/10	892	554	0/9	5/9	531	291	0/10	9/10	1,258	789	0/9	5/9
KIT-extrapolation_baseline	181	135	7/10	9/10	385	244	5/9	8/9	181	135	7/10	9/10	483	319	6/9	8/9
KIT-time_series_baseline	213	179	6/10	9/10	592	402	4/9	8/9	213	179	6/10	9/10	803	547	4/9	8/9
KITCOVIDhub-inverse_wis_ensemble	218	142	2/10	8/10	302	178	1/9	8/9	214	136	2/10	8/10	474	279	1/9	6/9
KITCOVIDhub-mean_ensemble	256	168	1/10	9/10	345	206	2/9	8/9	267	177	2/10	9/10	544	324	1/9	8/9
KITCOVIDhub-median_ensemble	251	165	2/10	8/10	381	251	2/9	7/9	242	159	3/10	8/10	543	330	2/9	8/9

Supplementary Table 3: Detailed summary of forecast evaluation for Poland (based on JHU data)

	Poland, cases															
	1 wk ahead inc				2 wk ahead inc				1 wk ahead cum				2 wk ahead cum			
Model	AE	WIS	$C_{0.5}$	$C_{0.95}$	AE	WIS	$C_{0.5}$	$C_{0.95}$	AE	WIS	$C_{0.5}$	$C_{0.95}$	AE	WIS	$C_{0.5}$	$C_{0.95}$
epiforecasts-EpiExpert	16,105	11,390	4/10	8/10	41,186	27,900	1/9	5/9	16,128	11,414	4/10	8/10	59,079	38,550	1/9	6/9
epiforecasts-EpiNow2	9,147	6,587	7/10	7/10	36,477	24,085	2/9	6/9	9,170	6,594	7/10	7/10	42,849	28,105	6/9	7/9
ICM-agentModel			2/4	4/4			0/3	1/3			1/4	3/4			0/3	2/3
ITWW-county_repro	19,441	16,205	1/10	4/10	37,152	30,879	2/9	2/9	18,519	15,278	1/10	3/10	57,467	46,975	1/9	3/9
LANL-GrowthRate	13,494*	8,719*	5/7	7/7	48,693*	27,686*	1/6	6/6	15,205	10,151	5/10	8/10	66,280	39,924	2/9	8/9
MIMUW-StochSEIR			3/5	4/5			2/4	2/4			2/5	4/5			1/4	2/4
MIT_CovidAnalytics-DELPHI	30,505*	22,627*	3/9	5/9	61,303*	46,360*	1/8	4/8								
MOCOS-agent1	15,732	11,468	1/10	4/10	32,235	26,111	1/9	3/9	15,732	11,468	1/10	4/10	46,214	35,642	1/9	3/9
SDSC_ISG-TrendModel	10,817								10,888							
USC-SIkJalpha	13,544		0/1	1/1	27,115				16,754		0/1	1/1	41,949			
KIT-baseline	31,605	20,001	5/10	9/10	55,931	37,396	2/9	6/9	31,676	20,036	5/10	9/10	87,597	59,162	2/9	6/9
KIT-extrapolation_baseline	18,333	11,754	7/10	10/10	55,685	34,091	3/9	8/9	18,311	11,752	7/10	10/10	77,269	45,119	3/9	9/9
KIT-time_series_baseline	22,502	14,455	5/10	10/10	60,704	38,643	4/9	9/9	22,480	14,452	5/10	10/10	85,192	53,310	4/9	9/9
KITCOVIDhub-inverse_wis_ensemble	14,191	9,103	5/10	8/10	37,174	25,943	3/9	6/9	14,325	8,838	5/10	8/10	50,096	33,472	2/9	6/9
KITCOVIDhub-mean_ensemble	13,849	8,879	4/10	9/10	37,831	24,819	2/9	6/9	14,511	8,727	5/10	8/10	50,731	32,216	2/9	7/9
KITCOVIDhub-median_ensemble	15,236	9,529	6/10	9/10	40,453	25,715	2/9	6/9	16,541	10,105	4/10	8/10	55,827	34,268	1/9	5/9

	Poland, deaths															
	1 wk ahead inc				2 wk ahead inc				1 wk ahead cum				2 wk ahead cum			
Model	AE	WIS	$C_{0.5}$	$C_{0.95}$	AE	WIS	$C_{0.5}$	$C_{0.95}$	AE	WIS	$C_{0.5}$	$C_{0.95}$	AE	WIS	$C_{0.5}$	$C_{0.95}$
epiforecasts-EpiExpert	303	186	3/10	9/10	625	399	2/9	7/9	303	186	3/10	9/10	911	571	2/9	8/9
epiforecasts-EpiNow2	343	240	5/10	7/10	1,066	758	3/9	5/9	344	240	5/10	7/10	1,439	1,003	3/9	5/9
ICM-agentModel	808*	715*	1/8	2/8	1,921*	1,272*	0/7	2/7	1,234*	770*	0/8	3/8	3,000*	1,904*	0/7	4/7
Imperial-ensemble2	379	229	6/10	7/10					351	200	6/10	7/10				
ITWW-county_repro	465	428	1/10	1/10	652	560	0/9	2/9	458	422	2/10	2/10	1,099	968	0/9	3/9
LANL-GrowthRate	236*	158*	4/7	7/7	383*	235*	4/6	6/6	236	154	4/10	9/10	655	420	3/9	8/9
MIMUW-StochSEIR			2/5	4/5			1/4	2/4			2/5	4/5			0/4	4/4
MIT_CovidAnalytics-DELPHI	467*	299*	2/9	7/9	621*	409*	1/8	6/8	552*	386*	2/9	7/9	990*	705*	1/8	4/8
MOCOS-agent1	205	153	8/10	10/10	393	253	5/9	8/9	205	153	8/10	10/10	533	359	7/9	9/9
SDSC_ISG-TrendModel	180								179							
USC-SIkJalpha	202		0/1	1/1	212				252		0/1	0/1	283			
KIT-baseline	504	305	5/10	10/10	882	578	2/9	6/9	503	304	5/10	10/10	1,365	896	2/9	5/9
KIT-extrapolation_baseline	412	274	6/10	8/10	995	700	5/9	7/9	411	274	6/10	8/10	1,422	974	4/9	7/9
KIT-time_series_baseline	528	333	8/10	10/10	1,343	853	5/9	8/9	529	333	8/10	10/10	1,909	1,206	5/9	8/9
KITCOVIDhub-inverse_wis_ensemble	207	138	7/10	10/10	476	300	4/9	9/9	231	151	7/10	10/10	683	433	4/9	8/9
KITCOVIDhub-mean_ensemble	227	147	7/10	10/10	558	349	4/9	9/9	250	158	6/10	10/10	793	500	4/9	9/9
KITCOVIDhub-median_ensemble	195	134	6/10	10/10	460	278	4/9	9/9	215	147	6/10	10/10	686	433	4/9	8/9

Supplementary Table 4: Summary of forecast evaluation for ensembles without plausibility checks of members (based on ECDC data)

Model	1 wk ahead inc AE	WIS	$C_{0.5}$	$C_{0.95}$	2 wk ahead inc AE	WIS	$C_{0.5}$	$C_{0.95}$	1 wk ahead cum AE	WIS	$C_{0.5}$	$C_{0.95}$	2 wk ahead cum AE	WIS	$C_{0.5}$	$C_{0.95}$
**Germany, cases**																
KITCOVIDhub-inverse_wis_ensemble_all	13,431	8,835	4/10	9/10	39,275	24,810	1/9	6/9	28,345	18,730	1/10	7/10	55,290	35,321	2/9	7/9
KITCOVIDhub-mean_ensemble_all	15,554	9,848	4/10	9/10	40,120	24,956	1/9	6/9	16,068	10,397	4/10	7/10	54,550	34,516	2/9	6/9
KITCOVIDhub-median_ensemble_all	11,240	7,959	6/10	9/10	36,823	23,838	3/9	7/9	13,511	9,593	6/10	7/10	51,242	35,038	2/9	6/9
**Germany, deaths**																
KITCOVIDhub-inverse_wis_ensemble_all	177	109	6/10	9/10	234	144	4/9	8/9	845	665	0/10	8/10	1,065	742	1/9	8/9
KITCOVIDhub-mean_ensemble_all	183	124	6/10	8/10	263	162	4/9	8/9	236	157	4/10	6/10	472	291	3/9	6/9
KITCOVIDhub-median_ensemble_all	185	129	6/10	8/10	332	217	3/9	7/9	196	132	4/10	7/10	434	270	3/9	7/9
**Poland, cases**																
KITCOVIDhub-inverse_wis_ensemble_all	12,100	8,065	4/10	9/10	36,692	23,049	3/9	7/9	11,951	7,300	5/10	10/10	44,256	28,051	2/9	8/9
KITCOVIDhub-mean_ensemble_all	11,788	7,847	4/10	9/10	37,031	22,548	2/9	8/9	11,649	7,076	5/10	10/10	43,910	27,625	3/9	9/9
KITCOVIDhub-median_ensemble_all	13,597	8,632	5/10	9/10	39,156	23,726	2/9	7/9	13,365	8,415	5/10	9/10	48,278	28,472	3/9	7/9
**Poland, deaths**																
KITCOVIDhub-inverse_wis_ensemble_all	188	141	6/10	10/10	467	295	5/9	9/9	384	241	2/10	9/10	656	406	5/9	9/9
KITCOVIDhub-mean_ensemble_all	204	147	7/10	9/10	593	353	4/9	9/9	220	154	7/10	9/10	802	485	3/9	9/9
KITCOVIDhub-median_ensemble_all	202	138	6/10	10/10	428	272	6/9	9/9	194	135	8/10	10/10	620	404	6/9	9/9

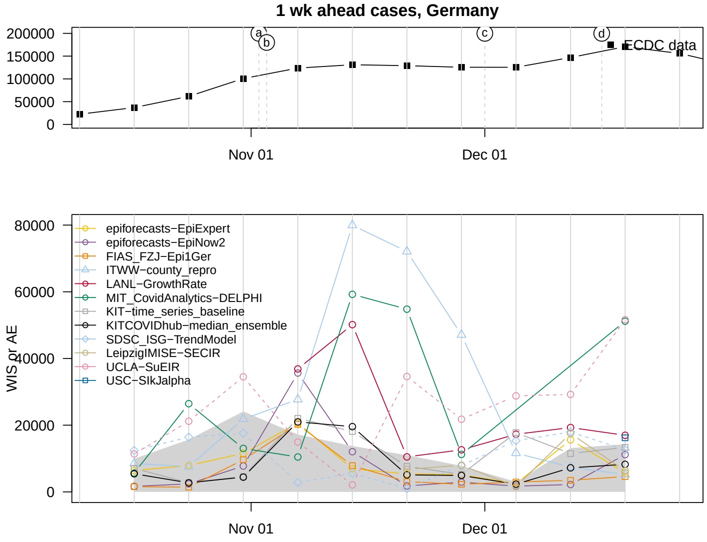

Supplementary Figure 5: WIS value or absolute error (of deterministic models) over time, for one-week-ahead case forecasts for Germany. Letters in circles represent events explained in Figure 1. As in Figure 7, the upper border of the light grey area represents the performance of the naïve baseline model `KIT-baseline`.

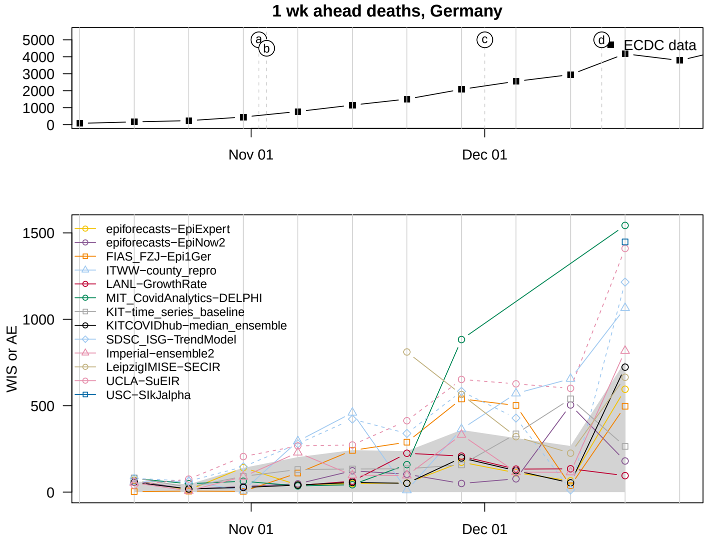

Supplementary Figure 6: WIS value or absolute error (of deterministic models) over time, for one-week-ahead death forecasts for Germany. Letters in circles represent events explained in Figure 1. As in Figure 7, the upper border of the light grey area represents the performance of the naïve baseline model `KIT-baseline`.

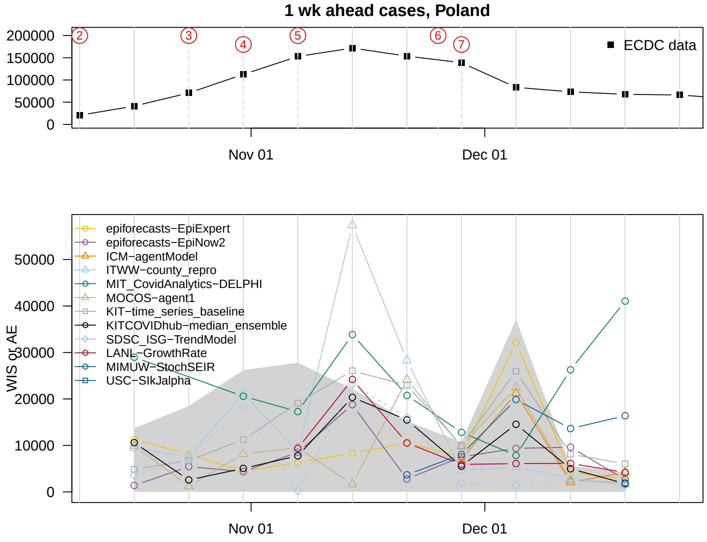

Supplementary Figure 7: WIS value or absolute error (of deterministic models) over time, for one-week-ahead case forecasts for Poland. Numbers in circles represent events explained in Figure 1. As in Figure 7, the upper border of the light grey area represents the performance of the naïve baseline model `KIT-baseline`.

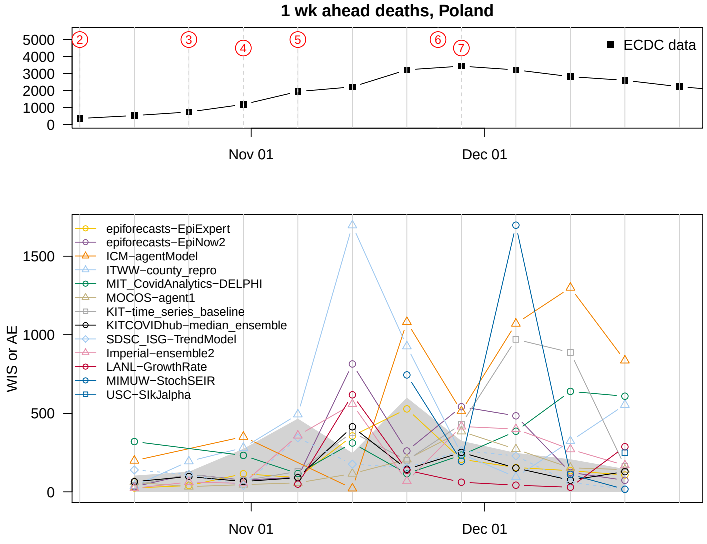

Supplementary Figure 8: WIS value or absolute error (of deterministic models) over time, for one-week-ahead death forecasts for Poland. Numbers in circles represent events explained in Figure 1. As in Figure 7, the upper border of the light grey area represents the performance of the naïve baseline model `KIT-baseline`.

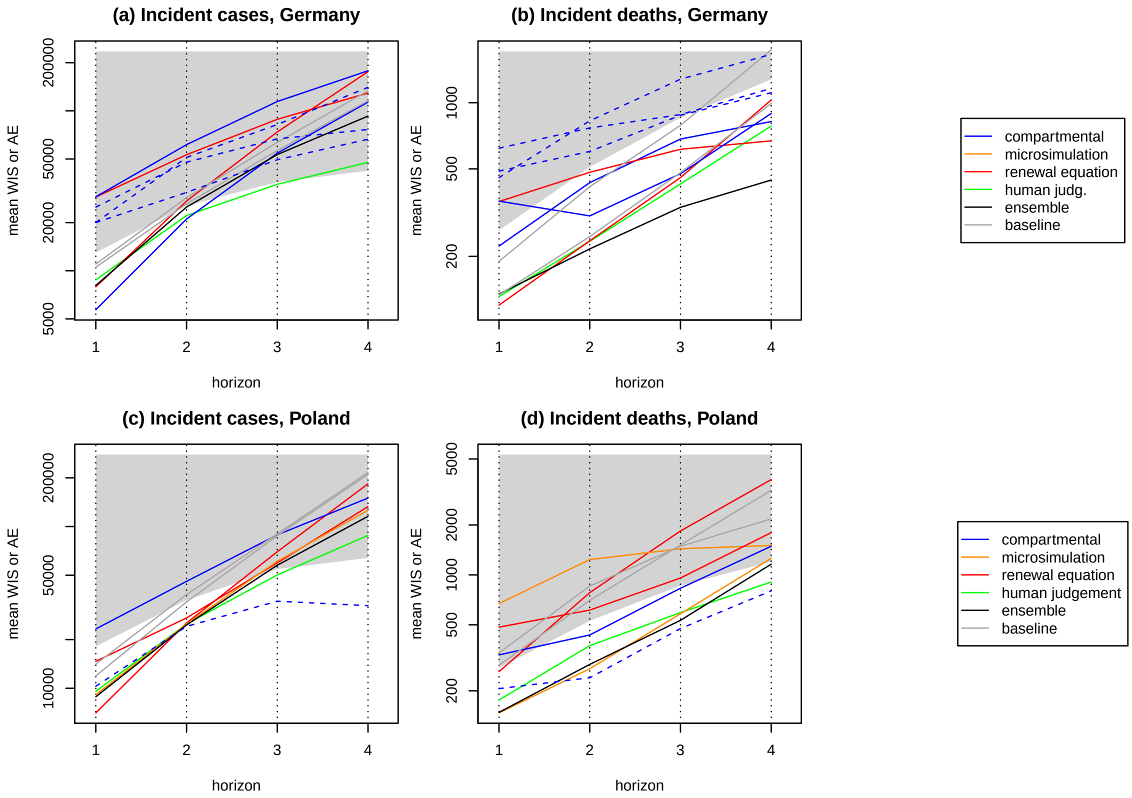

Supplementary Figure 9: Mean WIS or AE values by target and forecast horizon as in Figure 7. Lines are coloured according to the model categories introduced in Table 3. No clear patterns emerge apart from the fact that the ensemble model shows good relative performance for death forecasts.

Supplementary Note 8 Results for three- and four-week-ahead forecasts

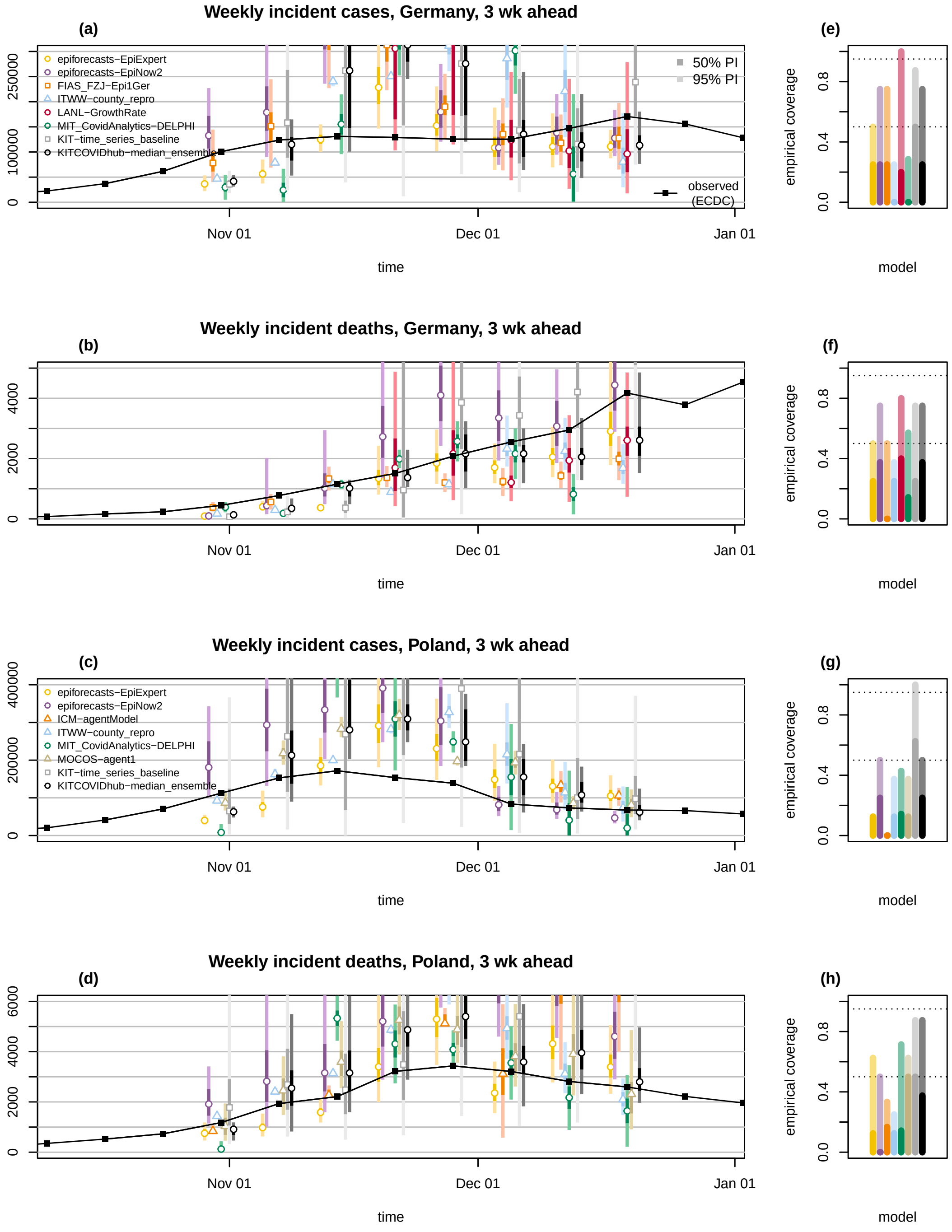

Supplementary Figure 10: **Three-week-ahead forecasts.** Three-week-ahead forecasts of incident cases and deaths in Germany (**a, b**) and Poland (**c, d**). Displayed are predictive medians, 50% and 95% prediction intervals (PIs). Coverage plots (**e–h**) show the empirical coverage of 95% (light) and 50% (dark) prediction intervals.

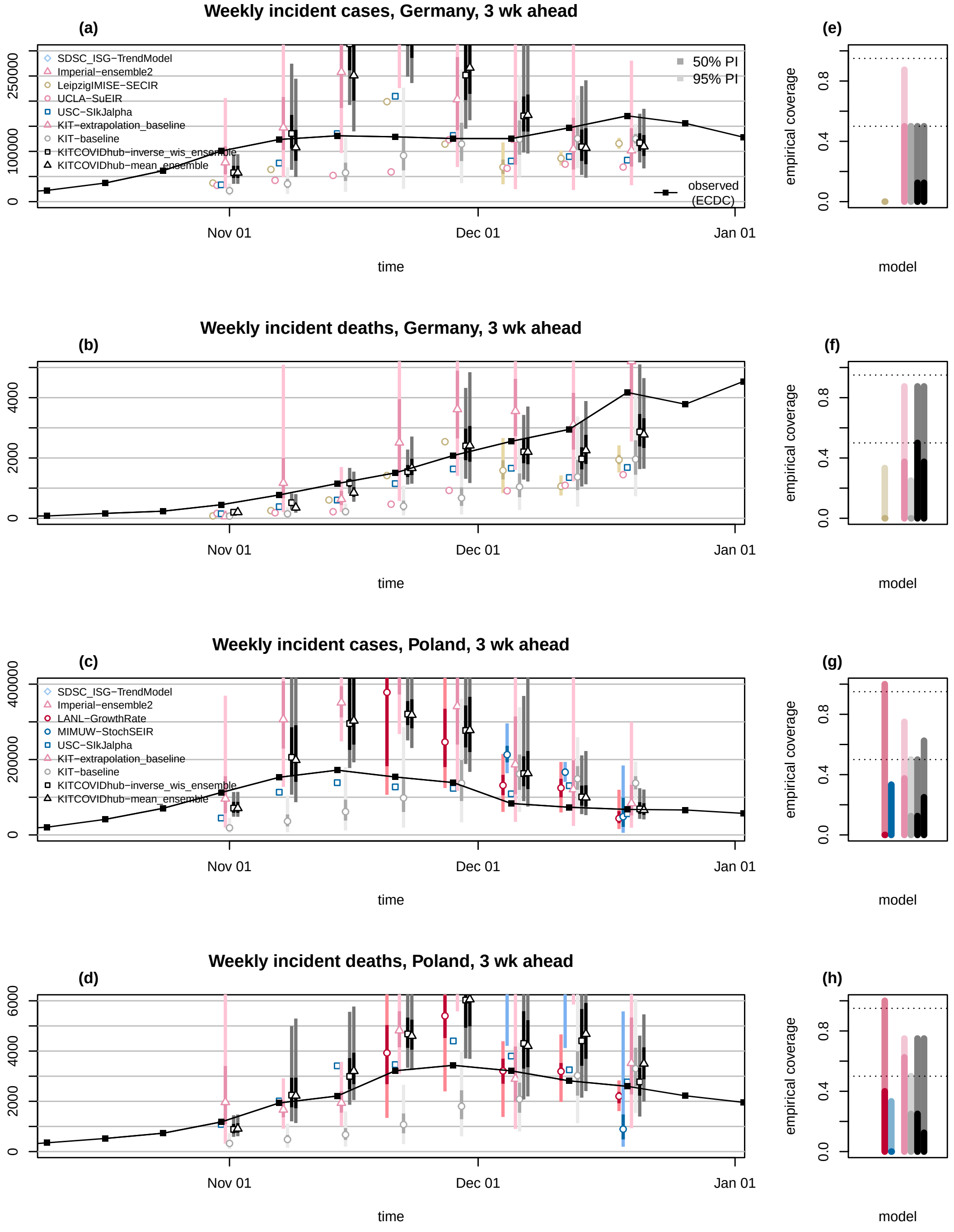

Supplementary Figure 11: **Additional three-week-ahead forecasts.** Three-week-ahead forecasts of incident cases and deaths in Germany (**a, b**) and Poland (**c, d**). Displayed are predictive medians, 50% and 95% prediction intervals (PIs) for models not shown in Figure 10. Coverage plots (**e–h**) show the empirical coverage of 95% (light) and 50% (dark) prediction intervals.

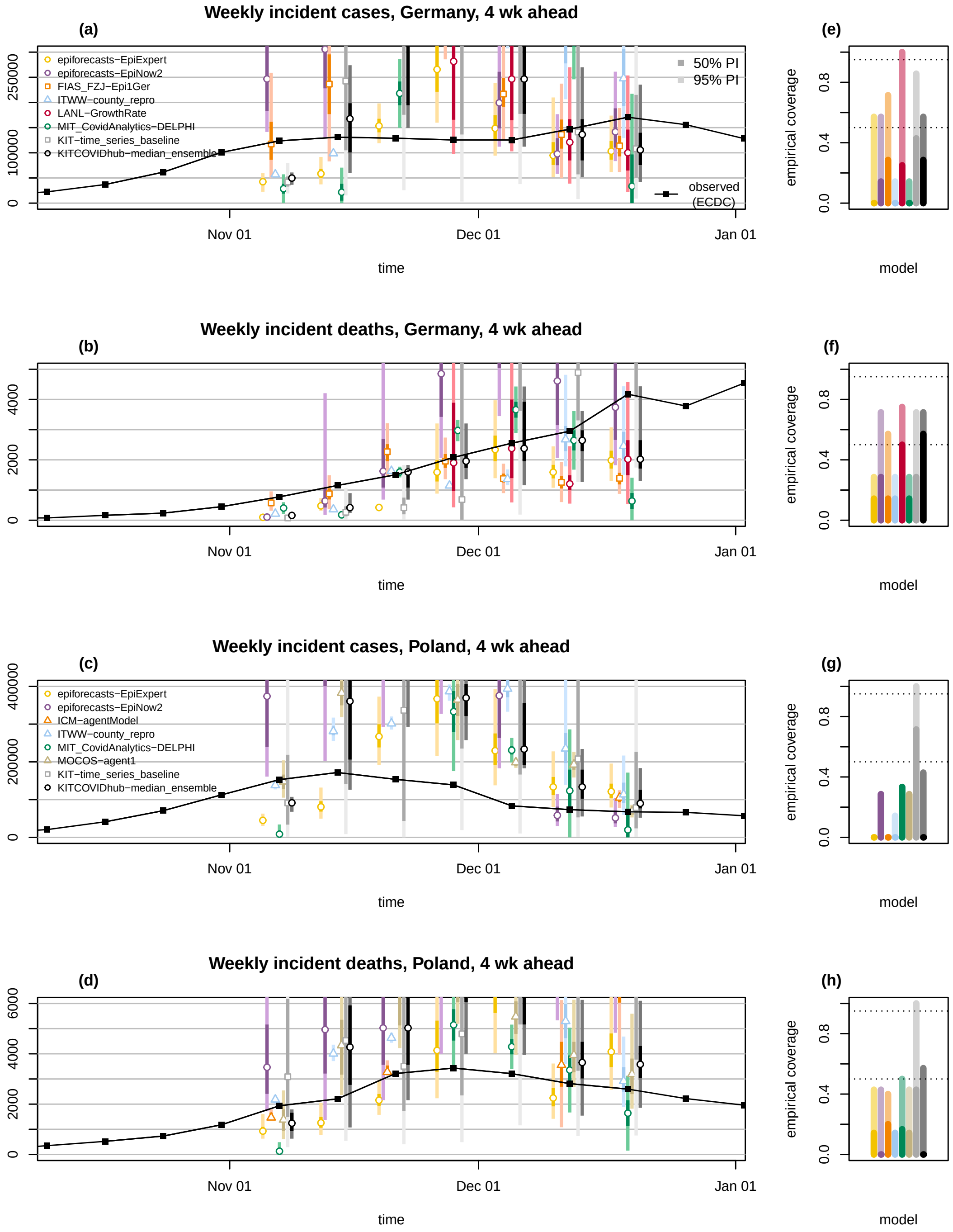

Supplementary Figure 12: **Four-week-ahead forecasts.** Four-week-ahead forecasts of incident cases and deaths in Germany (**a, b**) and Poland (**c, d**). Displayed are predictive medians, 50% and 95% prediction intervals (PIs). Coverage plots (**e–h**) show the empirical coverage of 95% (light) and 50% (dark) prediction intervals.

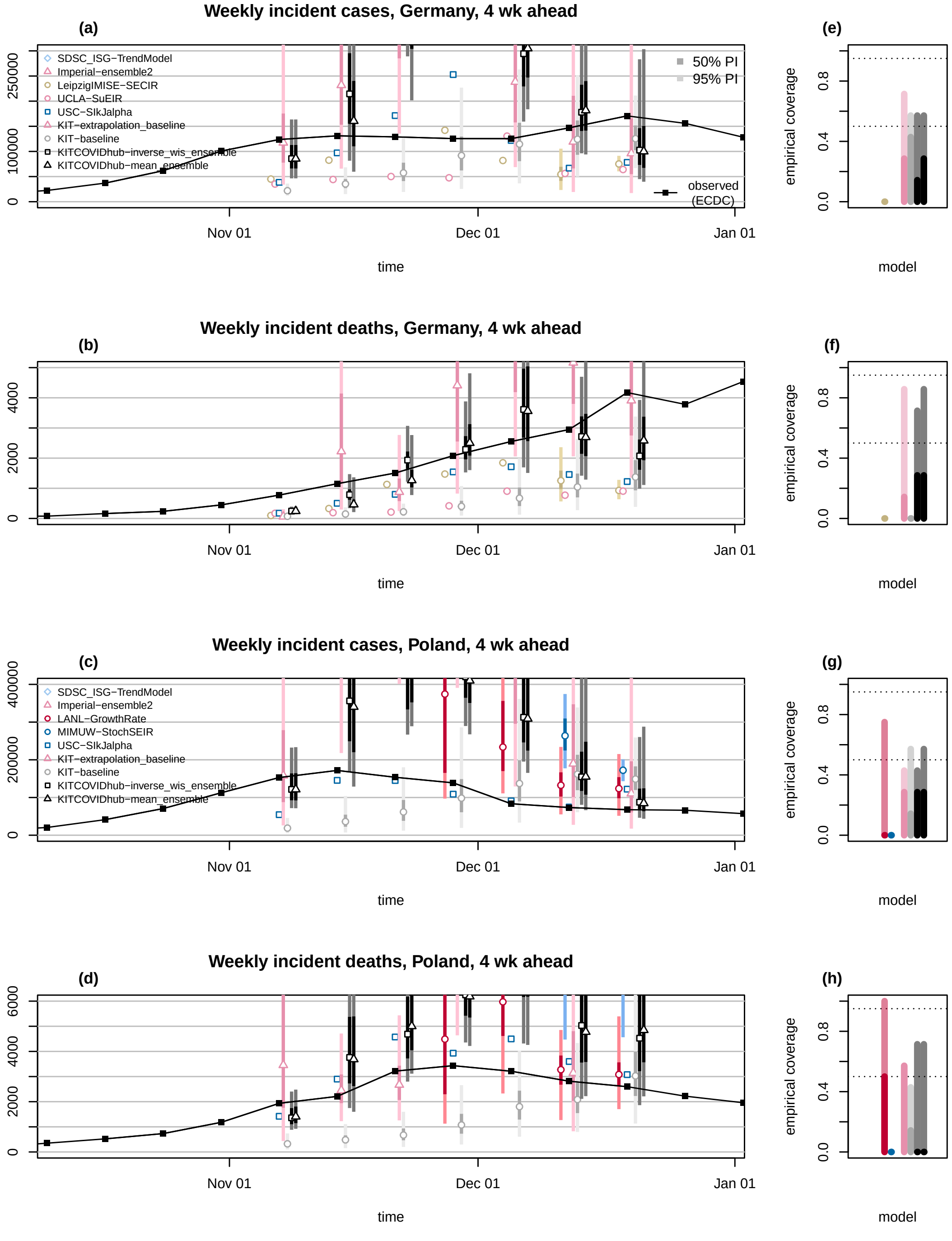

Supplementary Figure 13: **Additional four-week-ahead forecasts.** Four-week-ahead forecasts of incident cases and deaths in Germany (**a, b**) and Poland (**c, d**). Displayed are predictive medians, 50% and 95% prediction intervals (PIs) for models not shown in Figure 12. Coverage plots (**e–h**) show the empirical coverage of 95% (light) and 50% (dark) prediction intervals.

Supplementary Table 5: Detailed summary of forecast evaluation for Germany, 3 and 4 weeks ahead (based on ECDC data)

	Germany, cases															
	3 wk ahead inc				4 wk ahead inc				3 wk ahead cum				4 wk ahead cum			
Model	AE	WIS	$C_{0.5}$	$C_{0.95}$	AE	WIS	$C_{0.5}$	$C_{0.95}$	AE	WIS	$C_{0.5}$	$C_{0.95}$	AE	WIS	$C_{0.5}$	$C_{0.95}$
epiforecasts-EpiExpert	47,162	34,704	2/8	4/8	65,906	47,640	0/7	4/7	85,859	60,488	2/8	4/8	143,536	101,205	2/7	4/7
epiforecasts-EpiNow2	98,948	73,773	2/8	6/8	235,461	175,688	1/7	4/7	148,866	109,915	3/8	6/8	394,730	293,483	1/7	5/7
FIAS_FZJ-Epi1Ger	74,550	54,378	2/8	6/8	149,451	113,410	2/7	5/7	116,086	84,710	4/8	6/8	271,463	203,058	2/7	4/7
ITWW-county_repro	105,132	88,509	0/8	2/8	147,570	129,219	0/7	1/7	214,867	180,139	0/8	1/8	369,098	319,826	0/7	1/7
LANL-GrowthRate			1/5	5/5			1/4	4/4	218,206	152,213	2/8	5/8	328,115	246,869	2/7	4/7
LeipzigIMISE-SECIR	82,104		0/3	0/3	140,275		0/2	0/2	171,325	153,731	1/8	2/8	296,564	267,098	2/7	2/7
MIT_CovidAnalytics-DELPHI	139,142*	114,298*	0/7	2/7	210,370	178,016	0/7	1/7								
SDSC_ISG-TrendModel																
UCLA-SuEIR	66,768				76,415				132,374				198,546			
USC-SIkJalpha	49,446				66,436				96,902				140,716			
KIT-baseline	44,706	35,891	4/8	4/8	54,563	42,192	3/7	4/7	85,838	70,756	3/8	4/8	136,280	115,148	2/7	4/7
KIT-extrapolation_baseline	82,243	56,387	4/8	7/8	165,710	115,568	2/7	5/7	125,152	88,749	4/8	7/8	291,137	207,420	4/7	6/7
KIT-time_series_baseline	91,848	63,486	4/8	7/8	162,293	133,341	3/7	6/7	154,663	103,058	3/8	7/8	320,671	239,590	3/7	6/7
KITCOVIDhub-inverse_wis_ensemble	90,487	65,207	1/8	4/8	171,254	126,851	1/7	4/7	136,583	93,984	3/8	5/8	278,362	206,835	3/7	5/7
KITCOVIDhub-mean_ensemble	82,294	57,308	1/8	4/8	138,225	104,078	2/7	4/7	137,562	92,565	3/8	5/8	262,811	190,439	3/7	4/7
KITCOVIDhub-median_ensemble	79,238	53,265	2/8	6/8	129,400	92,703	2/7	4/7	125,538	89,344	2/8	5/8	252,946	191,986	3/7	4/7

	Germany, deaths															
	3 wk ahead inc				4 wk ahead inc				3 wk ahead cum				4 wk ahead cum			
Model	AE	WIS	$C_{0.5}$	$C_{0.95}$	AE	WIS	$C_{0.5}$	$C_{0.95}$	AE	WIS	$C_{0.5}$	$C_{0.95}$	AE	WIS	$C_{0.5}$	$C_{0.95}$
epiforecasts-EpiExpert	615	427	2/8	4/8	955	784	1/7	2/7	981	655	2/8	5/8	1,850	1,379	2/7	3/7
epiforecasts-EpiNow2	655	457	3/8	6/8	1,511	1,029	2/7	5/7	1,044	709	3/8	6/8	2,532	1,735	2/7	5/7
FIAS_FZJ-Epi1Ger	811	683	0/8	4/8	1,003	821	1/7	4/7	1,467	1,249	1/8	4/8	2,153	1,785	0/7	4/7
Imperial-ensemble2																
ITWW-county_repro	713	614	2/8	3/8	798	671	1/7	2/7	1,345	1,174	2/8	2/8	1,740	1,487	1/7	3/7
LANL-GrowthRate			2/5	4/5			2/4	3/4	1,364	1,098	2/8	4/8	2,238	2,006	2/7	3/7
LeipzigIMISE-SECIR	883		0/3	1/3	1,162		0/2	0/2	2,722	2,283	1/8	2/8	2,780	2,388	1/7	2/7
MIT_CovidAnalytics-DELPHI	593*	475*	1/7	4/7	1,039	893	1/7	2/7	1,211*	1,054*	0/7	1/7	2,201	2,018	0/7	0/7
SDSC_ISG-TrendModel																
UCLA-SuEIR	1,279				1,659				2,273				3,615			
USC-SIkJalpha	877				1,110				1,635				2,344			
KIT-baseline	1,218	855	0/8	2/8	1,608	1,277	0/7	0/7	2,186	1,584	0/8	0/8	3,538	2,947	0/7	0/7
KIT-extrapolation_baseline	752	481	3/8	7/8	1,526	992	1/7	6/7	1,252	799	3/8	7/8	2,666	1,731	1/7	6/7
KIT-time_series_baseline	1,092	789	2/8	6/8	1,712	1,739	2/7	5/7	1,727	1,259	3/8	7/8	2,934	2,802	2/7	5/7
KITCOVIDhub-inverse_wis_ensemble	440	274	4/8	7/8	704	471	2/7	5/7	810	499	2/8	6/8	1,259	881	4/7	4/7
KITCOVIDhub-mean_ensemble	488	297	3/8	7/8	676	433	2/7	6/7	880	557	3/8	6/8	1,301	837	3/7	5/7
KITCOVIDhub-median_ensemble	493	334	3/8	6/8	599	445	4/7	5/7	897	590	3/8	6/8	1,310	978	3/7	5/7

Supplementary Table 6: Detailed summary of forecast evaluation for Poland, 3 and 4 weeks ahead (based on ECDC data)

Poland, cases																
	3 wk ahead inc				4 wk ahead inc				3 wk ahead cum				4 wk ahead cum			
Model	AE	WIS	$C_{0.5}$	$C_{0.95}$	AE	WIS	$C_{0.5}$	$C_{0.95}$	AE	WIS	$C_{0.5}$	$C_{0.95}$	AE	WIS	$C_{0.5}$	$C_{0.95}$
epiforecasts-EpiExpert	69,036	50,186	1/8	1/8	114,361	88,135	0/7	0/7	123,420	83,370	1/8	5/8	236,085	163,096	0/7	2/7
epiforecasts-EpiNow2	100,091	69,829	2/8	4/8	257,145	183,720	2/7	2/7	146,121	96,446	3/8	6/8	422,386	291,126	2/7	4/7
ICM-agentModel			0/2	0/2			0/1	0/1			0/2	1/2			0/1	0/1
ITWW-county_repro	69,096	59,629	1/8	3/8	148,272	132,809	0/7	1/7	115,872	95,988	1/8	4/8	271,133	233,566	0/7	1/7
LANL-GrowthRate			0/5	5/5			0/4	3/4	147,981	85,560	1/8	7/8	278,420	163,238	1/7	5/7
MIMUW-StochSEIR			1/3	1/3			0/2	0/2			1/3	1/3			0/2	0/2
MIT_CovidAnalytics-DELPHI	113,802*	88,815*	1/7	3/7	177,706*	149,967*	2/6	2/6								
MOCOS-agent1	70,362	60,944	1/8	3/8	140,691	125,932	2/7	2/7	116,589	94,704	0/8	2/8	263,653	226,693	0/7	2/7
SDSC_ISG-TrendModel																
USC-SIkJalpha	34,543				32,410				71,142				110,078			
KIT-baseline	75,183	54,568	1/8	4/8	89,395	63,975	1/7	4/7	159,350	118,193	1/8	3/8	238,036	183,364	1/7	3/7
KIT-extrapolation_baseline	125,846	87,821	3/8	6/8	278,666	210,038	2/7	3/7	206,056	135,329	3/8	6/8	505,952	360,439	2/7	4/7
KIT-time_series_baseline	123,714	90,048	5/8	8/8	228,443	215,974	5/7	7/7	217,544	145,712	5/8	8/8	476,907	376,333	5/7	7/7
KITCOVIDhub-inverse_wis_ensemble	78,949	57,514	1/8	4/8	160,008	129,539	2/7	3/7	115,211	82,871	2/8	5/8	266,961	197,054	1/7	5/7
KITCOVIDhub-mean_ensemble	78,772	56,181	2/8	5/8	156,341	125,110	2/7	4/7	115,204	80,176	2/8	6/8	265,775	191,339	1/7	5/7
KITCOVIDhub-median_ensemble	74,351	57,640	2/8	4/8	144,957	115,659	0/7	3/7	126,339	77,669	1/8	5/8	275,236	171,357	1/7	4/7

Poland, deaths																
	3 wk ahead inc				4 wk ahead inc				3 wk ahead cum				4 wk ahead cum			
Model	AE	WIS	$C_{0.5}$	$C_{0.95}$	AE	WIS	$C_{0.5}$	$C_{0.95}$	AE	WIS	$C_{0.5}$	$C_{0.95}$	AE	WIS	$C_{0.5}$	$C_{0.95}$
epiforecasts-EpiExpert	901	592	1/8	5/8	1,329	906	1/7	3/7	1,828	1,135	1/8	6/8	3,171	2,035	1/7	4/7
epiforecasts-EpiNow2	2,642	1,840	0/8	4/8	5,317	3,757	0/7	3/7	4,316	2,977	1/8	4/8	9,641	6,722	0/7	4/7
ICM-agentModel	1,854*	1,437*	1/6	2/6	1,850*	1,505*	1/5	2/5	4,376*	2,847*	1/6	2/6	4,584*	3,275*	1/5	3/5
Imperial-ensemble2																
ITWW-county_repro	1,113	958	1/8	2/8	2,016	1,799	1/7	1/7	2,375	2,080	0/8	2/8	4,592	4,103	0/7	1/7
LANL-GrowthRate			2/5	5/5			2/4	4/4	1,517	938	4/8	7/8	2,984	1,956	3/7	6/7
MIMUW-StochSEIR			0/3	1/3			0/2	0/2			0/3	1/3			0/2	0/2
MIT_CovidAnalytics-DELPHI	1,122*	831*	1/7	5/7	1,851*	1,488*	1/6	3/6	2,076*	1,728*	1/7	1/7	3,899*	3,484*	0/6	1/6
MOCOS-agent1	940	583	4/8	5/8	1,883	1,259	1/7	3/7	1,450	890	5/8	7/8	3,497	2,072	3/7	4/7
SDSC_ISG-TrendModel																
USC-SIkJalpha	474				801				597				1,445			
KIT-baseline	1,208	862	2/8	4/8	1,544	1,201	1/7	3/7	2,368	1,768	1/8	4/8	3,986	3,226	1/7	2/7
KIT-extrapolation_baseline	1,995	1,514	5/8	6/8	4,093	3,234	4/7	4/7	3,537	2,561	4/8	6/8	7,690	5,969	3/7	4/7
KIT-time_series_baseline	2,235	1,487	4/8	7/8	3,084	2,176	3/7	7/7	3,799	2,505	5/8	7/8	5,787	3,872	4/7	7/7
KITCOVIDhub-inverse_wis_ensemble	1,039	649	2/8	6/8	2,205	1,447	0/7	5/7	1,668	1,069	4/8	8/8	3,850	2,467	2/7	5/7
KITCOVIDhub-mean_ensemble	1,165	710	1/8	6/8	2,232	1,461	0/7	5/7	1,978	1,242	3/8	6/8	4,109	2,653	1/7	5/7
KITCOVIDhub-median_ensemble	895	532	3/8	7/8	1,871	1,161	0/7	4/7	1,645	1,030	4/8	7/8	3,656	2,199	2/7	4/7
