# Supplementary material for: A pre-registered short-term forecasting study of COVID-19 in Germany and Poland during the second wave
Source: Nat Commun. 2021 Aug 27;12:5173. doi: 10.1038/s41467-021-25207-0 (PMC8397791; doi:10.1038/s41467-021-25207-0)
Supplement: Supplementary file 3 — Reporting Summary [file 41467_2021_25207_MOESM3_ESM.pdf]

nature research

Corresponding author(s): Johannes Bracher, Melanie Schienle

Last updated by author(s): Jul 27, 2021

Reporting Summary

Nature Research wishes to improve the reproducibility of the work that we publish. This form provides structure for consistency and transparency in reporting. For further information on Nature Research policies, see our Editorial Policies and the Editorial Policy Checklist.

Statistics

For all statistical analyses, confirm that the following items are present in the figure legend, table legend, main text, or Methods section.

n/a	Confirmed	
☐	☒	The exact sample size (*n*) for each experimental group/condition, given as a discrete number and unit of measurement
☒	☐	A statement on whether measurements were taken from distinct samples or whether the same sample was measured repeatedly
☒	☐	The statistical test(s) used AND whether they are one- or two-sided<br>*Only common tests should be described solely by name; describe more complex techniques in the Methods section.*
☒	☐	A description of all covariates tested
☒	☐	A description of any assumptions or corrections, such as tests of normality and adjustment for multiple comparisons
☐	☒	A full description of the statistical parameters including central tendency (e.g. means) or other basic estimates (e.g. regression coefficient) AND variation (e.g. standard deviation) or associated estimates of uncertainty (e.g. confidence intervals)
☒	☐	For null hypothesis testing, the test statistic (e.g. *F*, *t*, *r*) with confidence intervals, effect sizes, degrees of freedom and *P* value noted<br>*Give P values as exact values whenever suitable.*
☐	☒	For Bayesian analysis, information on the choice of priors and Markov chain Monte Carlo settings
☒	☐	For hierarchical and complex designs, identification of the appropriate level for tests and full reporting of outcomes
☒	☐	Estimates of effect sizes (e.g. Cohen's *d*, Pearson's *r*), indicating how they were calculated

Our web collection on statistics for biologists contains articles on many of the points above.

Software and code

Policy information about availability of computer code

Data collection: All forecasts were collected via a public GitHub repository (https://github.com/KITmetricslab/covid19-forecast-hub-de).

Data analysis: All analyses were performed using the R language for statistical computing, version 4.03. Codes to reproduce figures and tables are available at https://github.com/KITmetricslab/analyses_de_pl. Additional R packages used for the presented analyses are the following:
- forecast, version 8.12
- pals, version 1.7
- plotrix, version 3.8-1
- xtable, version 1.8-4

Participating teams used a wide range of additional software tools, documented in the respective references in Table 3. In particular this includes the following software packages mentioned in the manuscript or Supplementary Information:
- Stan Modeling Language, versions 2.24 and 2.25

and the R packages
- shiny, version 1.5.0
- EpiNow2, versions 1.3.0-1.3.2

For manuscripts utilizing custom algorithms or software that are central to the research but not yet described in published literature, software must be made available to editors and reviewers. We strongly encourage code deposition in a community repository (e.g. GitHub). See the Nature Research guidelines for submitting code & software for further information.

Data

Policy information about availability of data

All manuscripts must include a data availability statement. This statement should provide the following information, where applicable:

- Accession codes, unique identifiers, or web links for publicly available datasets
- A list of figures that have associated raw data
- A description of any restrictions on data availability

The forecast data generated in this study have been deposited in a GitHub repository (\url{https://github.com/KITmetricslab/covid19-forecast-hub-de}), with a stable Zenodo release available under accession code 4752079 (https://doi.org/10.5281/zenodo.4752079). This repository also contains all truth data used for evaluation. Details on how truth data were obtained can be found in Supplementary Section D. Forecasts can be visualized interactively at https://kitmetricslab.github.io/forecasthub/. Source data to reproduce Figures 1-7 are provided with this paper.

Field-specific reporting

Please select the one below that is the best fit for your research. If you are not sure, read the appropriate sections before making your selection.

- [x] Life sciences
- [ ] Behavioural & social sciences
- [ ] Ecological, evolutionary & environmental sciences

For a reference copy of the document with all sections, see nature.com/documents/nr-reporting-summary-flat.pdf

Life sciences study design

All studies must disclose on these points even when the disclosure is negative.

Sample size	We evaluate forecasts for 10 consecutive weeks, two countries and two different targets (deaths from COVID-19 and confirmed cases). This period was pre-specified in a study protocol and coincides with an epidemiologically meaningful temporal delimitation (given the arrival of new virus variants and vaccines in January 2021).
Data exclusions	No data were excluded from the forecast evaluations.
Replication	Computational replicability of the presented analyses is ensured by the availability of all forecast data, truth data and analysis codes in the two repositories mentioned in the data and code availability statements.<br>As our study is not an experimental study it was not possible to perform a full replication in the way one would do in a laboratory experiment. All teams generated and submitted forecasts in real time, a process which cannot be imitated post-hoc for an independent replication. All evaluations of the forecasts, however, are fully reproducible and do not involve any simulation or other stochastic/non-deterministic parts.
Randomization	This is not an experimental study, so no randomization could be applied. All participating teams attempted to forecast the course of the pandemic in real time and following the same instructions. No treatment of any sort which could have been randomized was applied.
Blinding	Examinators were not blinded in any way, but evaluation was done in an automated and fully reproducible way. There were no different treatment groups and performance evaluation did not involve any subjective measurements taken by researcher who could be biased by knowledge about treatment arms or similar aspects. This study bears very limited similarity to clinical or basic biomedical research and the problems addressed by blinding do not apply.

Reporting for specific materials, systems and methods

We require information from authors about some types of materials, experimental systems and methods used in many studies. Here, indicate whether each material, system or method listed is relevant to your study. If you are not sure if a list item applies to your research, read the appropriate section before selecting a response.

Materials & experimental systems

n/a	Involved in the study	
x		Antibodies
x		Eukaryotic cell lines
x		Palaeontology and archaeology
x		Animals and other organisms
x		Human research participants
x		Clinical data
x		Dual use research of concern

Methods

n/a	Involved in the study	
x		ChIP-seq
x		Flow cytometry
x		MRI-based neuroimaging
